# *catena*-Poly[[diformatocopper(II)]-μ-1,4-bis­(imidazol-1-yl)benzene]

**DOI:** 10.1107/S1600536811024019

**Published:** 2011-06-30

**Authors:** Bang Chen

**Affiliations:** a Key Laboratory of Synthetic and Natural Functional Molecule Chemistry (Ministry of Education), College of Chemistry & Materials Science, Northwest University, Xi’an 710069, People’s Republic of China

## Abstract

In the title compound, [Cu(CHO_2_)_2_(C_12_H_10_N_4_)], the Cu^II^ ion lies on an inversion center and is coordinated by two formate O atoms and two N atoms from two 1,4-bis­(imidazol-1-yl)phenyl ligands (*L*), forming a square-planar coordination environment. The linear mol­ecule *L* acts as a bidente bridging ligand, connecting the metal atoms into a chain along [101].

## Related literature

For background to coordination polymers containing imidazole-derived ligands, see: Cui *et al.* (2005 ▶); Jin *et al.* (2008 ▶); Li *et al.* (2009 ▶). 
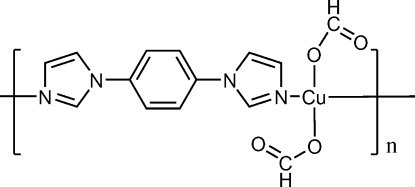


## Experimental

### 

#### Crystal data

[Cu(CHO_2_)_2_(C_12_H_10_N_4_)]
*M* *_r_* = 363.82
Monoclinic, 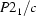

*a* = 8.0971 (16) Å
*b* = 10.426 (2) Å
*c* = 8.5723 (17) Å
β = 107.02 (3)°
*V* = 692.0 (2) Å^3^
*Z* = 2
Mo *K*α radiation
μ = 1.61 mm^−1^
*T* = 293 K
0.30 × 0.25 × 0.20 mm

#### Data collection

Rigaku Mercury CCD area-detector diffractometer
Absorption correction: multi-scan (*ABSCOR*; Higashi, 1995 ▶) *T*_min_ = 0.624, *T*_max_ = 0.725
6003 measured reflections
1204 independent reflections
1108 reflections with *I* > 2σ(*I*)
*R*_int_ = 0.026

#### Refinement

*R*[*F* ^2^ > 2σ(*F* ^2^)] = 0.025
*wR*(*F* ^2^) = 0.075
*S* = 1.11
1204 reflections
106 parameters
H-atom parameters constrained
Δρ_max_ = 0.40 e Å^−3^
Δρ_min_ = −0.39 e Å^−3^

### 

Data collection: *CrystalClear* (Rigaku/MSC, 2005 ▶); cell refinement: *CrystalClear*; data reduction: *CrystalClear*; program(s) used to solve structure: *SHELXS97* (Sheldrick, 2008 ▶); program(s) used to refine structure: *SHELXL97* (Sheldrick, 2008 ▶); molecular graphics: *SHELXTL* (Sheldrick, 2008 ▶); software used to prepare material for publication: *SHELXTL*.

## Supplementary Material

Crystal structure: contains datablock(s) I, global. DOI: 10.1107/S1600536811024019/rn2086sup1.cif

Structure factors: contains datablock(s) I. DOI: 10.1107/S1600536811024019/rn2086Isup2.hkl

Additional supplementary materials:  crystallographic information; 3D view; checkCIF report

## Figures and Tables

**Fig. 1.**
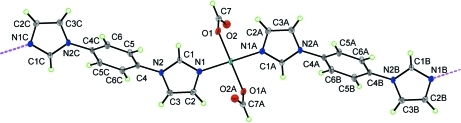
The molecular structure of (I). Displacement ellipsoids are drawn at the 30% probability level and H atoms are shown as small spheres of arbitrary radius (symmetry codes: A = 1-x, 1-y, -z; B = 1+x, y, -1+z; C = -x, 1-y, 1-z.).

**Fig. 2.**
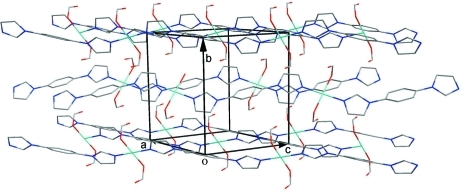
The crystal packing for (I).

## supplementary crystallographic information

### Comment

Imidazole has been extensively used in crystal engineering, and a large number of imidazole-containing flexible ligands have been extensively studied (Cui *et al.*, 2005; Jin *et al.*, 2008). However, to our knowledge, the research on imidazole ligands bearing rigid spacers is still less developed (Li *et al.*, 2009). For the title compound, the geometry of the Cu^II^ ion is bound by two imidazole rings of individual *L* ligands and two formate ions forming a square planar coordination environment (Fig. 1). Notably, as shown in Fig. 2, the four-coordinate Cu^II^ center is bridged by the linear ligand *L* to form an infinite one-dimensional chain along the [101] direction.

### Experimental

A mixture of CH_3_OH and H_2_O (1:1, 8 ml), as a buffer layer, was carefully layered over a solution of Cu(HCO_2_)_2_ in H_2_O (6 ml). Then a solution of 1,4-bis(imidazol-1-yl)phenyl (*L*, 0.06 mmol) in CH_3_OH (6 ml) was layered over the buffer layer, and the resultant reaction was left to stand at room temperature. After *ca.* three weeks, blue block single crystals appeared at the boundary. Yield: ~25% (based on *L*).

### Refinement

C-bound H atoms were positioned geometrically and refined in the riding-model approximation, with C—H = 0.93Å and *U*_iso_(H) = 1.2*U*_eq_ (C).

### Crystal data

**Table:**

[Cu(CHO_2_)_2_(C_12_H_10_N_4_)]	*F*(000) = 370
*M**_r_* = 363.82	*D*_x_ = 1.746 Mg m^−^^3^
Monoclinic, *P*2_1_/*c*	Mo *K*α radiation, λ = 0.71073 Å
*a* = 8.0971 (16) Å	Cell parameters from 2022 reflections
*b* = 10.426 (2) Å	θ = 2.0–27.9°
*c* = 8.5723 (17) Å	µ = 1.61 mm^−^^1^
β = 107.02 (3)°	*T* = 293 K
*V* = 692.0 (2) Å^3^	Block, blue
*Z* = 2	0.30 × 0.25 × 0.20 mm

### Data collection

**Table:**

Rigaku Mercury CCD area-detector diffractometer	1204 independent reflections
Radiation source: fine-focus sealed tube	1108 reflections with *I* > 2σ(*I*)
graphite	*R*_int_ = 0.026
Detector resolution: 9 pixels mm^-1^	θ_max_ = 25.0°, θ_min_ = 3.3°
ω scans	*h* = −9→9
Absorption correction: multi-scan (*ABSCOR*; Higashi, 1995)	*k* = −12→12
*T*_min_ = 0.624, *T*_max_ = 0.725	*l* = −10→10
6003 measured reflections	

### Refinement

**Table:**

Refinement on *F*^2^	Primary atom site location: structure-invariant direct methods
Least-squares matrix: full	Secondary atom site location: difference Fourier map
*R*[*F*^2^ > 2σ(*F*^2^)] = 0.025	Hydrogen site location: inferred from neighbouring sites
*wR*(*F*^2^) = 0.075	H-atom parameters constrained
*S* = 1.11	*w* = 1/[σ^2^(*F*_o_^2^) + (0.0452*P*)^2^ + 0.4041*P*] where *P* = (*F*_o_^2^ + 2*F*_c_^2^)/3
1204 reflections	(Δ/σ)_max_ < 0.001
106 parameters	Δρ_max_ = 0.40 e Å^−^^3^
0 restraints	Δρ_min_ = −0.39 e Å^−^^3^

### Special details

**Table:**

Geometry. All e.s.d.'s (except the e.s.d. in the dihedral angle between two l.s. planes) are estimated using the full covariance matrix. The cell e.s.d.'s are taken into account individually in the estimation of e.s.d.'s in distances, angles and torsion angles; correlations between e.s.d.'s in cell parameters are only used when they are defined by crystal symmetry. An approximate (isotropic) treatment of cell e.s.d.'s is used for estimating e.s.d.'s involving l.s. planes.
Refinement. Refinement of *F*^2^ against ALL reflections. The weighted *R*-factor *wR* and goodness of fit *S* are based on *F*^2^, conventional *R*-factors *R* are based on *F*, with *F* set to zero for negative *F*^2^. The threshold expression of *F*^2^ > σ(*F*^2^) is used only for calculating *R*-factors(gt) *etc*. and is not relevant to the choice of reflections for refinement. *R*-factors based on *F*^2^ are statistically about twice as large as those based on *F*, and *R*- factors based on ALL data will be even larger.

### Fractional atomic coordinates and isotropic or equivalent isotropic displacement parameters (Å^2^)

**Table:**

	*x*	*y*	*z*	*U*_iso_*/*U*_eq_	
Cu1	0.5000	0.5000	0.0000	0.01074 (16)	
O2	0.3615 (2)	0.73953 (14)	−0.05814 (18)	0.0215 (4)	
O1	0.55129 (17)	0.64376 (14)	0.15185 (16)	0.0154 (3)	
N2	0.1326 (2)	0.44066 (17)	0.24042 (19)	0.0116 (4)	
N1	0.3015 (2)	0.45187 (17)	0.0816 (2)	0.0128 (4)	
C7	0.4602 (3)	0.73795 (19)	0.0823 (3)	0.0168 (4)	
H7	0.4686	0.8130	0.1427	0.020*	
C4	0.0645 (3)	0.4692 (2)	0.3731 (2)	0.0123 (4)	
C3	0.0710 (2)	0.35192 (19)	0.1173 (2)	0.0142 (4)	
H3	−0.0238	0.2981	0.1032	0.017*	
C2	0.1769 (3)	0.35939 (19)	0.0218 (2)	0.0146 (4)	
H2	0.1673	0.3097	−0.0706	0.018*	
C6	−0.1121 (3)	0.4657 (2)	0.3490 (2)	0.0142 (4)	
H6	−0.1864	0.4419	0.2482	0.017*	
C1	0.2703 (3)	0.49981 (17)	0.2138 (3)	0.0112 (4)	
H1	0.3340	0.5647	0.2786	0.013*	
C5	0.1772 (3)	0.50202 (18)	0.5231 (3)	0.0144 (5)	
H5	0.2957	0.5025	0.5382	0.017*	

### Atomic displacement parameters (Å^2^)

**Table:**

	*U*^11^	*U*^22^	*U*^33^	*U*^12^	*U*^13^	*U*^23^
Cu1	0.0110 (2)	0.0136 (2)	0.0095 (2)	0.00028 (12)	0.00592 (16)	−0.00072 (12)
O2	0.0258 (8)	0.0209 (8)	0.0175 (8)	0.0000 (7)	0.0058 (6)	0.0008 (6)
O1	0.0144 (7)	0.0196 (8)	0.0142 (7)	0.0003 (6)	0.0073 (6)	−0.0034 (6)
N2	0.0118 (8)	0.0144 (9)	0.0107 (8)	0.0009 (7)	0.0063 (7)	0.0008 (7)
N1	0.0144 (8)	0.0131 (8)	0.0123 (8)	0.0014 (7)	0.0062 (7)	0.0000 (7)
C7	0.0202 (10)	0.0138 (10)	0.0205 (11)	−0.0035 (8)	0.0124 (9)	−0.0034 (9)
C4	0.0158 (10)	0.0131 (9)	0.0104 (9)	0.0010 (8)	0.0077 (8)	0.0029 (8)
C3	0.0139 (9)	0.0150 (10)	0.0140 (9)	−0.0027 (8)	0.0045 (8)	−0.0008 (8)
C2	0.0181 (10)	0.0150 (10)	0.0117 (9)	−0.0005 (8)	0.0059 (8)	−0.0030 (8)
C6	0.0128 (10)	0.0216 (10)	0.0080 (9)	−0.0016 (8)	0.0028 (8)	−0.0006 (8)
C1	0.0115 (11)	0.0137 (10)	0.0098 (10)	0.0003 (7)	0.0050 (9)	0.0012 (7)
C5	0.0101 (10)	0.0201 (12)	0.0143 (11)	−0.0004 (7)	0.0055 (9)	0.0014 (7)

### Geometric parameters (Å, °)

**Table:**

Cu1—O1^i^	1.9485 (14)	C7—H7	0.9300
Cu1—O1	1.9485 (14)	C4—C5	1.384 (3)
Cu1—N1^i^	1.9953 (17)	C4—C6	1.384 (3)
Cu1—N1	1.9953 (17)	C3—C2	1.350 (3)
O2—C7	1.235 (3)	C3—H3	0.9300
O1—C7	1.267 (3)	C2—H2	0.9300
N2—C1	1.351 (3)	C6—C5^ii^	1.390 (3)
N2—C3	1.382 (3)	C6—H6	0.9300
N2—C4	1.433 (3)	C1—H1	0.9300
N1—C1	1.329 (3)	C5—C6^ii^	1.390 (3)
N1—C2	1.381 (3)	C5—H5	0.9300
			
O1^i^—Cu1—O1	180.00 (8)	C5—C4—N2	119.09 (19)
O1^i^—Cu1—N1^i^	89.73 (7)	C6—C4—N2	119.75 (19)
O1—Cu1—N1^i^	90.27 (7)	C2—C3—N2	105.86 (17)
O1^i^—Cu1—N1	90.27 (7)	C2—C3—H3	127.1
O1—Cu1—N1	89.73 (7)	N2—C3—H3	127.1
N1^i^—Cu1—N1	180.0	C3—C2—N1	109.86 (17)
C7—O1—Cu1	107.51 (12)	C3—C2—H2	125.1
C1—N2—C3	107.92 (16)	N1—C2—H2	125.1
C1—N2—C4	124.60 (17)	C4—C6—C5^ii^	119.3 (2)
C3—N2—C4	127.48 (17)	C4—C6—H6	120.4
C1—N1—C2	106.15 (17)	C5^ii^—C6—H6	120.4
C1—N1—Cu1	125.33 (14)	N1—C1—N2	110.19 (18)
C2—N1—Cu1	128.41 (13)	N1—C1—H1	124.9
O2—C7—O1	126.19 (19)	N2—C1—H1	124.9
O2—C7—H7	116.9	C4—C5—C6^ii^	119.6 (2)
O1—C7—H7	116.9	C4—C5—H5	120.2
C5—C4—C6	121.1 (2)	C6^ii^—C5—H5	120.2
			
O1^i^—Cu1—O1—C7	−151.1 (3)	C1—N2—C3—C2	1.1 (2)
N1^i^—Cu1—O1—C7	93.37 (13)	C4—N2—C3—C2	−179.90 (19)
N1—Cu1—O1—C7	−86.63 (13)	N2—C3—C2—N1	−0.7 (2)
O1^i^—Cu1—N1—C1	168.60 (17)	C1—N1—C2—C3	0.1 (2)
O1—Cu1—N1—C1	−11.40 (17)	Cu1—N1—C2—C3	176.50 (14)
N1^i^—Cu1—N1—C1	−7(100)	C5—C4—C6—C5^ii^	1.2 (3)
O1^i^—Cu1—N1—C2	−7.16 (17)	N2—C4—C6—C5^ii^	−177.73 (18)
O1—Cu1—N1—C2	172.84 (17)	C2—N1—C1—N2	0.6 (2)
N1^i^—Cu1—N1—C2	177 (100)	Cu1—N1—C1—N2	−175.95 (12)
Cu1—O1—C7—O2	−2.2 (3)	C3—N2—C1—N1	−1.1 (2)
C1—N2—C4—C5	−35.7 (3)	C4—N2—C1—N1	179.88 (17)
C3—N2—C4—C5	145.4 (2)	C6—C4—C5—C6^ii^	−1.2 (3)
C1—N2—C4—C6	143.3 (2)	N2—C4—C5—C6^ii^	177.73 (18)
C3—N2—C4—C6	−35.6 (3)		

Symmetry codes: (i) −*x*+1, −*y*+1, −*z*; (ii) −*x*, −*y*+1, −*z*+1.
